# Babassu (*Attalea glassmanii* Zona) Nut Oil Is More Effective than Olive Oil for Treating Ischemia-Reperfusion Injury

**DOI:** 10.1155/2020/2525871

**Published:** 2020-09-21

**Authors:** Fábio França Silva, Daniela Signorelli Balthazar, Thauany Hellmann, Joaquim Silva Sales, Gyl Eanes Barros Silva, Fátima Zely Garcia de Almeida Cyrino, Maria Célia Pires Costa, Raquel Maria Trindade Fernandes, Marcos Antonio Custódio Neto da Silva, Maria do Carmo Lacerda Barbosa, Wanderson Romão, Bruno Gomes de Oliveira, Boniek Gontijo Vaz, Eliete Bouskela, Maria do Desterro Soares Brandão Nascimento

**Affiliations:** ^1^Postgraduate Program in Biotechnology (RENORBIO), Empresa Brasileira de Serviços Hospitalares, Laboratory of Immunofluorescence and Electron Microscopy, University Hospital, Federal University of Maranhão, R. Barão de Itapary 227, Centro, 65020-070 São Luís, MA, Brazil; ^2^Laboratory of Clinical and Experimental Research in Vascular Biology (BioVasc), Reitor Haroldo Lisboa da Cunha Pavilion, State University of Rio de Janeiro, Rua São Francisco Xavier 524, Térreo, 20550-013 Rio de Janeiro, RJ, Brazil; ^3^Laboratory of Macromolecular and Natural Products, Department of Chemistry, State University of Maranhão, Education and Exact and Natural Sciences Center, Paulo VI University Campus, São Cristóvão, P.O. Box 09, 65067-320 São Luís, MA, Brazil; ^4^Postgraduate Program in Adult Health (PPGSAD), Biological and Health Sciences Center, Federal University of Maranhão, Bacanga University Campus, Avenida dos Portugueses s/n, Block 3, Room 3A, 65085-580 São Luís, MA, Brazil; ^5^Department of Chemistry and Biology (CECEN), State University of Maranhão, Education and Exact and Natural Sciences Center, Paulo VI University Campus, São Cristóvão, P.O. Box 09, 65067-320 São Luís, MA, Brazil; ^6^Postgraduate Program in Clinical Medicine, Medical Sciences School, State University of Campinas, Rua Vital Brazil 251, Zeferino Vaz University City, Barão Geraldo, Campinas, SP, Brazil; ^7^Federal Institute of Espírito Santo (IFES), Ministro Salgado Filho Avenue, S/No-Soteco Neighborhood, 29106-010 Vila Velha, ES, Brazil

## Abstract

**Background:**

Cardiovascular disease (CVD) is the leading cause of death in Western civilizations. The type of fatty acid which makes up the diet is related to the cardiovascular morbimortality and the formation of atheromas. Populations with high consumption of oils and fats have a higher number of deaths from CVD.

**Purpose:**

In the present study, the objective was to comparatively analyze the microcirculatory effects of unrefined babassu oil with olive oil in microcirculation and liver of male hamsters of the species *Mesocricetus auratus*, checking the permeability to macromolecules after ischemia-reperfusion (I/R) without and with topical application of histamine 5 × 10^−6^ M. This is an experimental study, using as model the hamster's cheek pouch, which was prepared for intravital microscopy. The hamsters were divided into seven groups and orally treated for 14 days, twice a day (at 8 AM and 4 PM), orally received treatments in the following doses: unrefined babassu oil (BO) 0.02 mL/dose (group BO-2), 0.06 mL/dose (group BO-6), and 0.18 mL/dose (BO-18 group); extra virgin olive oil (OI) 0.02 mL/dose (group OI-2), 0.06 mL/dose (group OI-6), and 0.18 mL/dose (OI-18 group); and mineral oil (MO) 0.18 mL/dose (MO-18 group). The observations were made on the 15th day on the hamsters' cheek pouch; the increase of vascular permeability induced by I/R with and without histamine application was evaluated, and in the liver the biological material was collected aseptically then fixed in 10% buffered formalin.

**Results:**

Microcirculatory analyses showed a significant reduction in the number of leaks after I/R with and without the topical use of histamine in animals treated with unrefined BO 0.06 mL/dose (BO-6) and 0.18 mL/dose (BO-18) compared to animals treated with OI. The BO group (*p* < 0.001) presented a dose-response relationship for decreasing leaks after I/R with and without topical use of histamine. Histological liver analyses showed no fat deposition changes in any of the treatment groups. Phytochemical analyses evidenced a chemical compound (C_31_H_60_NO_8_) in unrefined BO but not in OI.

**Conclusions:**

This experiment demonstrates the protective effect of unrefined BO on the microcirculatory system and its greater dose effect than that of OI. Finding a chemical compound (C_31_H_60_NO_8_) that is present in BO but not in OI opens the possibility of investigating whether this chemical compound was responsible for the protective effect on membrane permeability.

## 1. Background

Cardiovascular diseases (CVDs) are characterized by functional changes in the cardiac system and are responsible for 30% of deaths worldwide, of which more than 80% occur in middle- and low-income countries due to eating habits associated with modifiable (hyperlipidemia, smoking, alcoholism, hyperglycemia, obesity, sedentarism, and contraceptive use) and nonmodifiable risk factors (family history, age, sex, and race). Populations that consume a higher quantity of fat present a higher mortality rate due to CVDs [1–4].

The influence of dietary fatty acids on risk factors for CVD and on lipid and lipoprotein plasma concentrations has been widely studied. The lipidic composition of the diet is essential to prevent and treat several diseases and to maintain the body's biochemical and physiological status [5, 6].

Research on the association between dietary habits, diet composition, and protection against diseases is underway. An example is the growing interest in the Mediterranean diet and the relevant role it plays in preventing inflammation and CVDs. Clinical, epidemiological, and experimental evidence suggests that the extra virgin oil-rich Mediterranean diet, specifically extra virgin olive oil (OI), reduces the incidence of oxidative stress, chronic inflammation, and immune diseases such as cancer, atherosclerosis, and CVDs [7].

The unrefined babassu oil (BO) extracted from babassu palms of the Arecaceae family, genera *Orbignya* and *Attalea*, is part of the diet of several quilombola communities that survive on extractive agriculture [8–10].

Approximately 65% of the weight of the almond is BO, which contains triglycerides, small amounts of free fatty acids, phospholipids, pigments, sterols, tocopherols, and traces of other substances and metals [10, 11].

BO is rich in lauric acid, with the concentration of lauric acid in the oil being above 40%. Lauric acid is very important in the food industry for resisting nonenzymatic oxidation and, unlike other saturated fats, it has a low and well-defined fusion temperature. It can be used in the preparation of special fats used in confectioneries, ice creams, margarine, and cocoa butter substitutes. It is also used in the cosmetics industry owing to its physical properties and oxidation resistance property [8, 12–14].

The objective of this study was to compare the microcirculatory vascular effects of unrefined BO from *Attalea glassmanii* Zona and extra virgin OI on the liver and plasma using the cheek pouches of male hamsters (*Mesocricetus auratus*) as a model.

## 2. Materials and Methods

### 2.1. Animals

This is an experimental study, using the diverticulum of the hamster oral vestibule (Jugal bag) as a model, in which it was prepared for intravital microscopy according to Dulling [15] and modified by Svenjö [16] and Bouskela and Gramp [17] for the study of microcirculation and the liver for histological study.

This was an experimental study using 70 male hamsters (*Mesocricetus auratus*, Botucatu, São Paulo, SP, Brazil) (7 to 10 weeks old) weighing 122 to 146 g. The animals were kept in the Laboratory of Clinical and Experimental Research in Vascular Biology, Rio de Janeiro State University, in a temperature- and humidity-controlled environment, under a 12/12 h light/dark cycle with *ad libitum* water intake and on an autoclaved standard diet (Nuvital, Nuvilab, Curitiba, Brazil).

The animals were divided into seven groups (*n* = 10/groups) and treated twice a day for 14 days using unrefined babassu oil (BO) at 0.02 mL/dose (BO-2 group), 0.06 mL/dose (BO-6 group), and 0.18 mL/dose (BO-18 group); extra virgin olive oil (OI) at 0.02 mL/dose (OI-2 group), 0.06 mL/dose (OI-6 group), and 0.18 mL/dose (OI-18 group); and mineral oil (MO) at 0.18 mL/dose (MO-18 group).

The oils were administrated by gavage, and the doses were adjusted for animal body weight.

These seven groups were then subdivided: six animals from each group were used for the analysis of I/R-induced vascular permeability with and without the use of histamine. Meanwhile, four animals from each group were used for histological liver studies for fat deposition and to analyze the blood biochemical parameters of triglyceride plasma concentrations, total cholesterol, high-density lipoprotein (HDL), low-density lipoprotein (LDL), and very-low-density lipoprotein (VLDL). Sterile MO was used as a negative control as it is considered safe [18] and for its poor absorption in the gastrointestinal tract [19].

### 2.2. BO

The almonds were collected from babassu plantations in the municipality of Lago dos Rodrigues, MA, Brazil. The oil was extracted using the continuous extraction method at the Chemistry Laboratory of the State University of Maranhão, São Luís, MA, Brazil, using a Soxhlet Fat and Lipid Extractor (Foss Tecator Soxtec HT 6; Fisher Scientific, Pittsburg, PA, USA) and a heater (Tecnal, Piracicaba, Brazil) previously heated at 150°C for 60 min. Hexane was used as a solvent [20]; Instituto Adolfo [21, 22].

### 2.3. OI

The study used extra virgin OI of the commercial brand Andorinha® bought in the retail trade in São Luís, MA, Brazil. This oil had the following characteristics: acidity ≤ 0.50%, peroxide index ≤ 20.00 meqO_2_/kg, ultraviolet specific extinction 270 nm ≤ 0.22, and delta *K* ≤ 0.01, 232 nm ≤ 2.50.

### 2.4. Microcirculatory Studies by Intravital Microscopy

At day 15, the animals were anesthetized with an intraperitoneal injection of sodium pentobarbital 0.02 mL∙100 g body weight (Pentobarbital Sodium, Sanofi, Paris, France, 60 mg/mL) and maintained with intravenous *α*-chloralose (2.5% solution) in the femoral vein. A tracheal cannula (PE 190, Becton Dickinson and Sparks, MD, USA) was inserted to facilitate spontaneous ventilation. Body temperature was maintained at 36.5°C using a heated plate and monitored using a rectal thermometer. Cheek pouches were prepared for intravital microscopy as previously described in the literature [23–25]. The cheek pouch was everted and placed on the microscope plate for analysis. An area of approximately 1 cm^2^ was prepared for the study of microvascular permeability by intravital microscopy.

The cheek pouch was continuously irrigated using a HEPES ((4-(2-hydroxyethyl)-1-piperazineethanesulfonic acid) bicarbonate-buffered saline solution and 95% N_2_ and 5% CO_2_ to maintain pH 7.4 under low oxygen tension. Thirty minutes after the preparation phase, each hamster received an intravenous injection (25 mg∙100 g body weight) of fluorescein isothiocyanate (FITC-dextran, molecular weight 150.000, TdB Consultancy, Uppsala, Sweden). Using an inflatable tourniquet to compress the proximal region of the everted pouch, total ischemia was achieved and then maintained for 30 min [23].

Macromolecular permeability was quantified by counting the number of leaks in the preparation. Leak sites were defined as fluorescence points near a postcapillary venule [23, 24]. Only animals that presented less than ten leaks after the FITC-dextran marker injection were considered as part of this study. Sixty minutes after reperfusion, topical histamine (5 × 10^−6^ M) (Sigma, St. Louis, MO, USA) was applied continuously for 5 min. The number of leaks was evaluated 2, 5, 10, and 15 min after the use of histamine. Statistical analysis considered the number of leaks10 min after reperfusion and 5 min after the use of histamine.

### 2.5. Histological and Biochemical Studies

Blood was drawn from the femoral vein, placed in test tubes without anticoagulant, and centrifuged at 3000 rpm for 10 min in a table centrifuge. The sera obtained by centrifugation were stored in Eppendorf microtubes and kept frozen at −80°C. Blood parameters of plasma concentrations of triglycerides, total cholesterol, HDL, LDL, and VLDL were determined using Labtest commercial kits (Labtest Diagnóstica S.A., Brazil). Concentrations were obtained by spectrophotometry using a semiautomatic SX- 3000M Sinnowa Brasil® biochemical analyzer.

After blood collection, the animals were euthanized using a lethal dose of sodium pentobarbital 0.2 mL∙100 g body weight (120 mg/kg). Necropsy started immediately after death and the liver was aseptically collected, macroscopically analyzed, fixed in 10% buffered formalin with pH 7.2, processed, and embedded in paraffin. Five 1-*μ*m-thick histological sections were placed on glass slides cleaned with alcohol-ether solution. The slides were stained using the hematoxylin-eosin (HE) method for routine analysis of histological changes. Histological sections were visualized under a 40x microscope, digitized using a Leica DFC340FX microcamera connected to a Leica DM5000B microscope, and analyzed using the Leica image processing and analysis software.

### 2.6. Fourier Transform Ion Cyclotron Resonance Mass Spectrometry (FTICR-MS)

The phytochemical analysis of unrefined BO and OI samples was conducted by Fourier Transform Ion Cyclotron Resonance Mass Spectrometry (FTICR-MS), using an FTICR-MS spectrometer (9.4T, Bruker Daltonics Solarix, Bremen, Germany). The spectra were obtained in negative-ion mode in a mass-to-charge ratio (m/z) range of 150–1200. Electrospray mass ionization (ESI) source conditions were 0.5 bar nebulizer gas pressure, 3.9 kV capillary voltage, and 180°C capillary transfer temperature. Ion accumulation time was 0.25 s. Each spectrum was acquired by the accumulation of 100 scans. The spectra were obtained in high resolution (4 M). FTICR-MS injection contained 1 mg of the extract solubilized in 1 mL of methanol P.A. (Vetec Química Fina Ltd., Brazil) and 4 *μ*L of NH4OH P.A. solution (Vetec Química Fina Ltd., Brazil). Mass spectra were acquired and processed using the Compass Data Analysis software (Bruker Daltonics, Bremen, Germany). The structural compound formulas were obtained from the ChemSpider database (http://www.chemspider.com).

### 2.7. Statistical Analysis

The Stata/SE 9.0 software for Windows (Stata Corporation, College Station, TX, USA) was used for all statistical analyses. The Shapiro–Wilk test was used to verify the normality of the studied variables. Mean was used as a measure of central tendency and standard deviation as a measure of dispersion. The ANOVA analysis of variance was used to compare groups, followed by the Tukey *post hoc* test for *p* < 0.05. A significance level of 5% (*p* < 0.05) was used for all tests. Histological analysis was qualitative.

### 2.8. Ethical Aspects

All procedures were approved by the Animal Experiments Committee (Protocol CEUA no. 039/2015) according to the International Standards for the Use of Animals in Biomedical Research (*Consejo de Organizaciones Internacionales de las Ciencias Médicas*, 1990) and the Federal Law no. 6,638 (May 8, 1979), which establishes norms for the didactic-scientific practice of animal vivisection.

## 3. Results

### 3.1. Body Weight Analysis of Animals in Different Treatment Groups

In this study, 70 hamsters were allocated in three experimental groups, using MO as a negative control and OI and unrefined BO as treatments. Body weight analysis showed no statistical difference (Table 1). Regarding body weight information, the three groups of extra virgin olive OI and the three groups of babassu oil were grouped.

### 3.2. I/R-Induced Microvascular Leak with and without the Use of Histamine in the Different Treatment Groups

In this study, the hamsters were orally administered MO, OI, and unrefined BO and responded to I/R.

The results showed a significant reduction of mean leak values during reperfusion without the topical use of histamine in animals treated with unrefined BO at 0.06 mL/dose (BO-6) and 0.18 mL/dose (BO-18) when compared to animals treated with OI at 0.06 mL/dose (OI-6) and 0.18 mL/dose (OI-18) and MO in the control group (MO-18) (Table 2).

The use of topical histamine was statistically significant for 0.06 mL/dose and 0.18 mL/dose in the BO, OI, and MO groups (Table 3).

A dose effect was observed in the BO group, decreasing I/R-induced microvascular leak with and without the topical use of histamine (*p* < 0.0001).

### 3.3. Biochemical Study of Plasma Total Cholesterol, HDL-Cholesterol, LDL-Cholesterol, VLDL, and Triglyceride Levels

The studied treatments did not change plasma total cholesterol, HDL-cholesterol, LDL-cholesterol, VLDL, and triglyceride levels and had no statistical significance (Tables 4 and 5).

### 3.4. Histological Study of the Liver

**Figure 1:**
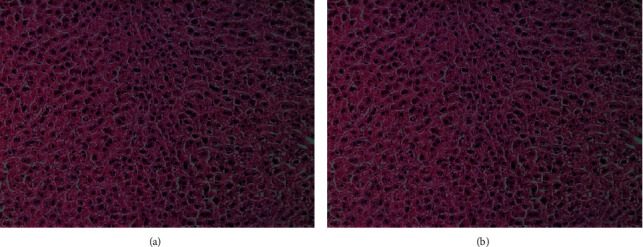
(a) Histological section of untreated normal liver parenchyma, hematoxylin/eosin stained, 40x magnification. (b) Histological section of treated liver parenchyma showing no lipid vacuoles (macrovesicular steatosis), hematoxylin/eosin stained. Similar histological pattern for all groups treated with babassu oil, extra virgin olive oil, or mineral oil; 40x magnification.

Histological analysis of the liver showed no fat deposition changes that could characterize steatosis in the groups treated with OI, unrefined BO, and MO **(NC)** (Figures 1(a) and 1(b)). The liver analysis did not show any toxicity signal.

### 3.5. Phytochemical Study by Mass Spectrometry

**Figure 2:**
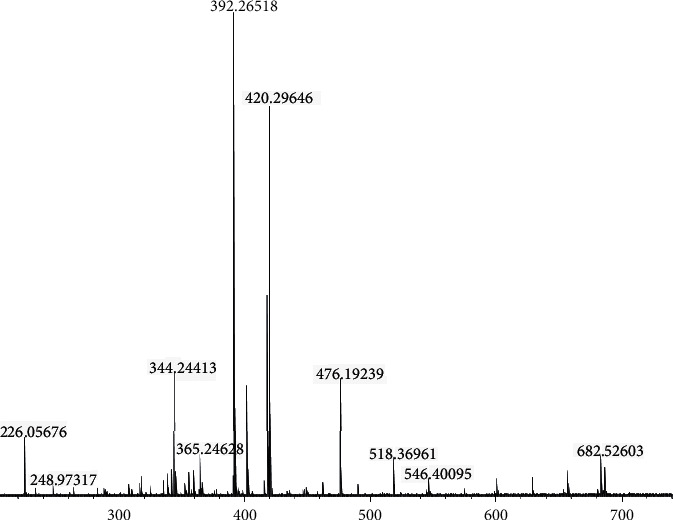
Mass spectrum of unrefined babassu oil.

Mass spectrometry showed the presence of a chemical compound (C31H60NO8) in unrefined BO (Table 6 and Figure 2) but not in OI and the presence of C37H70NO8 in unrefined BO and OI, respectively (Table 7). However, compound degradation byproducts were present only in BO.

## 4. Discussion

Oils and fats are predominantly fatty acid and glycerol triesters called triacylglycerols. The type of fatty acid consumed in the diet may increase fat deposition in adipose tissue, change lipid profile, and, consequently, influence the development of chronic noncommunicable diseases [26].

Some studies reported that diets including large amounts of saturated fatty acids (SFA) result in higher fat accumulation when compared to diets rich in monounsaturated (MUFAs) and polyunsaturated (PUFAs) fatty acids. Some studies relate PUFA consumption with greater weight gain and obesity induction in hamsters and rats when compared with SFA consumption [27–30].

An experiment with hamsters reported that an oil mixture consisting of 60% MUFAs and equal amounts of PUFAs to SFA, with a blend of high-fat soybean and canola oils, can prevent body weight gain and body fat accumulation. This can reduce insulin concentrations and increase liver lipolytic enzyme activities [31].

Diets rich in oils and fats are proven to be one of the factors inducing obesity and related comorbidities, such as hypertension, diabetes mellitus, hyperlipidemia, and CVDs. The type of oil and fat influences metabolic functions and leads to weight and body composition changes. It can also change blood levels of circulating lipids, which can lead to atherosclerosis, a change in medium- and large-caliber arteries, which causes intimal layer thickening, elasticity loss, and subsequent calcification [32, 33].

Lipid profile changes and the development of certain comorbidities associated with oil and fat consumption such as diabetes mellitus, steatosis, and others are directly related to the amount of oils and fats consumed, consumption duration, and the fatty acid composition of these oils and fats [34].

Lauric acid is considered a “healthier” saturated fat for being a medium-chain triglyceride (MCT) which is easily absorbed by the body. Some health benefits of lauric acid are its excellent antibacterial, antiviral, and antifungal actions, helping to combat various pathogenic microorganisms. In addition to these properties, some studies reported a thermogenic property that helps body and abdominal fat loss in addition to weight loss [35, 36].

SFA tend to increase low-density lipoprotein-cholesterol (LDL-c) levels by suppressing low-density lipoprotein-receptors (LDL-r) and decreasing plasma clearance of these particles, which were not observed in our study because the major compound that transforms BO into a saturated oil is lauric acid. Furthermore, SFA facilitate cholesterol entry into LDL particle membranes due to their rectilinear chemical structure, contributing to an increase in LDL-c levels. FA effects seem to be limited to chain lengths between 10 and 18 carbons, with myristic (C14:0) and palmitic (C16:0) acids being more atherogenic [37, 38]. However, increased LDL levels were not seen in our study.

OI is considered to be one of the largest sources of MUFAs in the human diet, with 80% oleic acid in its composition [39, 40].

This high content of oleic acid makes OI less susceptible to oxidation than the PUFAs that predominate in other oils, making OI more stable. The high proportion of oleic acid lowers LDL-c levels and increases HDL-c levels in the blood [41–44].

The replacement of SFA by MUFAs in the diet, particularly oleic acid, has a protective effect against the development of coronary artery disease (CAD). Diets rich in MUFAs reduce LDL-c, oxidize LDL plasma concentrations, and increase smooth muscle cell proliferation without modifying HDL-c concentrations. Reduced LDL-c is most likely due to increased LDL-r expression, increasing plasma lipoprotein removal. MUFAs are also less susceptible to free radical oxidation than PUFAs due to a reduced number of double bonds in their chemical structure [44–46].

In this study, microcirculatory results demonstrated that unrefined BO is superior to OI in decreasing the number of I/R-induced leaks with and without the use of topical histamine. The study also revealed a dose effect in the BO group, decreasing I/R-induced microvascular leaks with and without the use of topical histamine (*p* < 0.0001).

These data corroborate the results of a study that first studied the effect of unrefined BO on microcirculation compared with MO, reporting that macromolecular leak values after I/R injury were significantly lower in animals treated with unrefined BO [47].

A study used hamsters' cheek pouches as a model to evaluate the protective effect of n-3 fatty acid supplementation on microcirculation and reported macromolecular permeability and microvascular reactivity after I/R. They found that fish oil supplementation significantly reduces the number of macromolecular leaks after I/R. After I/R, the animals supplemented with OI presented arteriolar constriction and the animals supplemented with fish oil presented dilation followed by a return to the diameter observed in the preischemia phase. These results suggest that the type of fatty acid used in the diet plays an important role in regulating endothelial function and inflammatory processes [48].

A dose-dependent effect was observed in our study in the group treated with unrefined BO. One study reported that dietary supplementation with fish oil reduces the number of macromolecular leaks after I/R with increased dose-dependent inhibition of I/R-induced macromolecular permeability [48]. The differences in the number of leaks between groups treated with different doses of fish oil decreased as the daily supplementation dose increased. This can be explained by the fact that n-3 fatty acids produce a series of biologically inactive prostaglandins, thromboxane (series 3), and leukotrienes (series 5) [49, 50].

Unrefined BO contains minor components such as sterols, tocopherols, and oleic acid, which have been investigated for microcirculation protective effects [11, 12, 50].

FTICR-MS was used for the phytochemical study. Developed in the early 1970s, it is considered the most complex type of mass analyzer. Its analytical power is the result of its versatility and a unique combination of features such as very-high-resolution power and mass accuracy. This ensures a definite determination of the chemical formula of the produced ions, n-type analysis capability (MS) to determine molecular structure, and easy adaptation to many types of external ionization sources [51–54].

Phytochemical analysis identified the presence of a chemical compound (C_31_H_60_NO_8_) in unrefined BO but not in OI and the presence of C_37_H_70_NO_8_ in both samples. Compound degradation byproduct C_31_H_60_NO_8_ was present only in BO, but no information on the chemical compound could be found. However, the compound C_37_H_70_NO_8_ was cataloged in the MOLBASE website (http://www.molbase.com/), one of the largest integrated chemistry platforms.

The presence of these compounds in unrefined BO but not in OI may be related to the reduced number of macromolecular leaks in the I/R models used in this research. Further studies on the action of these compounds on microcirculation physiology are needed.

This study shows the protective effect of unrefined BO on the microcirculatory system with and without the topical use of histamine and its superior dose effect when compared with OI, supporting the inclusion of BO in the functional food category as a vascular protector against CVDs.

The discovery of a chemical compound (C_31_H_60_NO_8_) that is only present in BO and not in OI opens the possibility of future investigation into whether this compound was responsible for the reduced number of macromolecular leaks in the I/R models used in this study. These are new and important data that may contribute to the classification of BO as a functional food in the market of edible oils and as a drug for the treatment of CVD in the future. However, further studies are necessary to confirm this hypothesis.

## Figures and Tables

**Table 1:** Body weight of animals in the experimental groups treated with mineral oil (NC), olive oil, and unrefined babassu oil using 0.02, 0.06, and 0.18 mL/dose twice a day.

Treatment	Initial weight (g)	Final weight (g)	*p* ^*∗*^
Mineral oil (*n* = 10)	143.4 ± 10.1	140.6 ± 10.8	**0.5193**
Olive oil (*n* = 30)	147.8 ± 9.5	145.5 ± 9.2	**0.6524**
Unrefined babassu oil (*n* = 30)	144.1 ± 10.3	143.7 ± 10.8	**0.8884**
*p* ^*∗*^	**0.2621**	**0.3679**	

Results are expressed as mean ± standard deviation. Analysis of variance complemented by the Tukey test with ^*∗*^*p* < 0.05 showed no differences between means.

**Table 2:** Mean number of leaks in the experimental permeability/ischemia/reperfusion study without the use of histamine for the animal groups treated with mineral oil (NC), olive oil, and unrefined babassu oil using 0.02, 0.06, and 0.18 mL/dose twice a day.

Treatment (dose)	Groups without the use of histamine			
	MO group (*n* = 6)	OI group (*n* = 18)	BO group (*n* = 18)	*p* value
0.02 mL/dose	—	127.5 + 23.9	117.3 + 3.2	**0.5272**
0.06 mL/dose	0	120.7 + 17.0^a^	104.8 + 3.0^*∗∗*^^a^	**0.0137**
0.18 mL/dose	122.8 + 1.7^a^	116.7 + 14.1^a^	90.2 + 4.2^*∗∗∗*^^a^	**<0.0001**
*p* ^*∗*^	—	**0.6159**	**<0.0001**	

No animals were used for these doses. ^a^Statistical difference between groups using the same dose (Tukey's test). ^*∗*^*p* < 0.05, ^*∗∗*^*P* < 0.01, and ^*∗∗∗*^*P* < 0.001.

**Table 3:** Mean number of leaks in the experimental permeability/ischemia/reperfusion study with the use of histamine for the animal groups treated with mineral oil (NC), olive oil, and unrefined babassu oil using 0.02, 0.06, and 0.18 mL/dose twice a day.

Treatment (dose)	Groups with the use of histamine			
	MO group (*n* = 6)	OI group (*n* = 18)	BO group (*n* = 18)	*p* value
0.02 mL/dose	—	238.5 + 82.2	219.0 + 5.4	**0.7002**
0.06 mL/dose	—	206.5 + 43.9^a^	200.7 + 7.0^a^^*∗*^	**0.0456**
0.18 mL/dose	241.2 + 17.4^a^	190.3 + 57.9^a^	185.5 + 10.4^a^^*∗*^	**0.0280**
*p* ^*∗*^	—	**0.5692**	**<0.0001**	

No animals were used for these doses. ^a^Statistical difference between groups using the same dose (Tukey's test). ^*∗*^*p* < 0.05, ^*∗∗*^*P* < 0.01, and ^*∗∗∗*^*P* < 0.001.

**Table 4:** Effect of unrefined babassu oil using 0.02, 0.06, and 0.18 mL/dose twice a day on the serological parameters of male *Mesocricetus auratus* hamsters.

Lipid fraction	0.02 mL/dose (*n* = 4)	0.06 mL/dose (*n* = 4)	0.18 mL/dose (*n* = 4)	0.18 mL/dose (NC) (*n* = 4)	*p* value
Serum					
Total cholesterol	66.80 ± 14.05	69.83 ± 8.96	88.16 ± 34.93	63.00 ± 7.32	**0.1597**
HDL-cholesterol	18.50 ± 5.96	19.17 ± 8.91	20.33 ± 8.98	20.33 ± 5.72	**0.9658**
LDL-cholesterol	21.50 ± 9.05	25.67 ± 5.01	23.67 ± 8.38	20.50 ± 6.16	**0.6304**
VLDL	26.80 ± 2.45	25.00 ± 2.99	44.16 ± 27.16	22.17 ± 5.94	**0.0587**
Triglycerides	134.0 ± 12.25	125.0 ± 14.95	220.78 ± 137.26	110.85 ± 29.68	**0.0572**

Results are presented as mean ± standard deviation, different lowercase letters in the same column indicate significant differences (*p* < 0.05), and different uppercase letters in the same column indicate significant differences (*p* < 0.05). ^*∗*^ANOVA, after Tukey's test. The reference total cholesterol value for hamsters is 50–120 mg/dL.

**Table 5:** Effect of extra virgin olive oil using 0.02, 0.06, and 0.18 mL/dose twice a day on the serological parameters of male *Mesocricetus auratus* hamsters.

Lipid fraction	0.02 mL/dose (*n* = 4)	0.06 mL/dose (*n* = 4)	0.18 mL/dose (*n* = 4)	0.18 mL/dose (NC) (*n* = 4)	*p* value
Serum					
Total cholesterol	65.73 ± 4.63	66.90 ± 9.16	67.78 ± 16.82	63.00 ± 7.32	**0.8740**
HDL-cholesterol	20.17 ± 5.81	20.00 ± 7.69	21.17 ± 6.77	20.33 ± 5.72	**0.9895**
LDL-cholesterol	19.83 ± 4.54	21.17 ± 10.76	25.17 ± 6.46	20.50 ± 6.16	**0.6027**
VLDL	25.73 ± 3.51	25.73 ± 3.51	21.45 ± 9.04	22.17 ± 5.94	**0.5486**
Triglycerides	128.67 ± 17.56	128.67 ± 17.56	107.23 ± 45.21	110.85 ± 29.68	**0.5276**

Results are presented as mean ± standard deviation, different lowercase letters in the same column indicate significant differences (*p* < 0.05), and different uppercase letters in the same column indicate significant differences (*p* < 0.05). ^*∗*^ANOVA, after Tukey's test. The reference total cholesterol value for hamsters is 50–120 mg/dL.

**Table 6:** Analysis of chemical components present in the unrefined babassu oil sample identified by mass spectrometry using an FTICR-MS spectrometer.

m/z (exp.)	Molecular formula (M-H)	DBE	Error (ppm)
226.05676	C_6_H_12_NO_8_	1	0.35
339.23284	C_23_H_31_O_2_	8	0.33
344.24413	C_18_H_34_NO_5_	2	0.34
353.2121	C_23_H_29_O_3_	9	0.33
392.26518	C_19_H_38_NO_7_	1	0.49
402.22843	C_23_H_32_NO_5_	8	0.41
420.29646	C_21_H_42_NO_7_	1	0.52
462.30703	C_23_H_44_NO_8_	2	0.45
476.19239	C_24_H_30_NO_9_	10	0.46
490.33831	C_25_H_48_NO_8_	2	0.48
518.36961	C_27_H_52_NO_8_	2	0.45
546.40095	C_29_H_56_NO_8_	2	0.36
574.43231	C_31_H_60_NO_8_	2	0.23
600.44789	C_33_H_62_NO_8_	3	0.34
628.47913	C_35_H_66_NO_8_	3	0.41
656.51032	C_37_H_70_NO_8_	3	0.57
682.52603	C_39_H_72_NO_8_	4	0.46

**Table 7:** Analysis of chemical components present in the olive oil sample identified by mass spectrometry using an FTICR-MS spectrometer.

m/z (exp.)	Molecular formula (M-H)	DBE	Error (ppm)
226.05676	C_6_H_12_NO_8_	1	0.36
344.24418	C_18_H_34_NO_5_	2	0.20
381.13029	C_17_H_21_N_2_O_8_	8	0.13
392.26519	C_19_H_38_NO_7_	1	0.48
420.29649	C_21_H_42_NO_7_	1	0.44
440.11964	C_19_H_22_NO_11_	9	0.43
454.13543	C_20_H_24_NO_11_	9	0.12
476.19248	C_24_H_30_NO_9_	10	0.26
656.51052	C_37_H_70_NO_8_	3	0.25
682.52603	C_39_H_72_NO_8_	4	0.46

## Data Availability

The data used to support the findings of this study are available from the corresponding author upon request.

## Abbreviations

BioVasc: Laboratory of Vascular Biology, State University of Rio de Janeiro
BO: Babassu oil
CAD: Coronary artery disease
CVD: Cardiovascular diseases
FTICR-MS: Fourier transform ion cyclotron resonance mass spectrometry
HDL: High-density lipoprotein
LDL-c: Low-density lipoprotein-cholesterol
LDL-r: Low-density lipoprotein-receptors
MCT: Medium-chain triglyceride
MUFA: Monounsaturated fatty acids
OI: Olive oil
PUFAs: Polyunsaturated fatty acids
VLDL: Very-low-density lipoprotein.
